# An invertebrate-specific and immune-responsive microRNA augments oyster haemocyte phagocytosis by targeting CgIκB2

**DOI:** 10.1038/srep29591

**Published:** 2016-07-12

**Authors:** Hao Chen, Zhi Zhou, Hao Wang, Lingling Wang, Weilin Wang, Rui Liu, Limei Qiu, Linsheng Song

**Affiliations:** 1 Key Laboratory of Experimental Marine Biology, Institute of Oceanology, Chinese Academy of Sciences, Qingdao 266071, China; 2 University of Chinese Academy of Sciences, Beijing 100049, China; 3 Key Laboratory of Mariculture & Stock enhancement in North China’s Sea, Ministry of Agriculture, Dalian Ocean University, Dalian 116023, China

## Abstract

Nuclear factor (NF)-κB pathway is an evolutionally conserved pathway in activating immune response, in which IκBs can repress the activation. In the present study, cgi-miR-2d, an invertebrate-specific microRNA, was proved to regulate CgIκB2 expression and haemocyte phagocytosis during bacterial infection in oyster *Crassostrea gigas*. The expression of cgi-miR-2d was significantly up-regulated after *Vibrio splendidus* challenge, while CgIκB2 transcripts decreased. Significant decreases in both luminescence and CgIκB2 3′UTR level was observed after transfection of cgi-miR-2d in CgIκB2 3′UTR luciferase reporter assay. CgIκB2 mRNA level decreased significantly (0.51-fold of control group, *p* < 0.05) in gain-of-function assay of cgi-miR-2d *in vivo* while it increased markedly (1.27-fold, *p* < 0.05) when cgi-miR-2d was repressed (0.10-fold, *p* < 0.01). A significant increase of haemocyte phagocytosis rate was observed in cgi-miR-2d overexpression group (*p* < 0.01), consistent with results in CgIκB2 knock-down group (*p* < 0.01). Moreover, the apoptosis rate of haemocytes was found significantly declined (28.57%, *p* < 0.01) in gain-of-function assay of cgi-miR-2d. Together, those results not only depicted the functional conservation of miR-2d family in anti-apoptosis of oysters but also highlighted its interaction with phagocytosis by modulating NF-κB pathway, which might dedicate critically to the well-balance of host immune response.

Phagocytosis of immunocytes is an essential process in host immune response against invaded pathogens^1^. As a highly integrated cellular activity, it comprises a series of events, including pathogen recognition by cell surface receptors, intracellular signal transduction, cytoskeletal rearrangement, particle engulfment, microbial lysogenesis and antigen presentation to other immunocytes^2^. The phagocytosis against infiltrated pathogens, as found, can be robustly activated after challenge and strictly modulated afterward by diverse genes or pathways, keeping the well-balance of host immune system^3^. Among those regulators, nuclear factor-κB (NF-κB) family^4^ is the most important ones as a global activator of phagocytosis-related genes^5^.

In mammals, the NF-κB family is mainly composed by a family of five structurally related transcriptional factors, including NF-κB1 (p105/p50), NF- κB2 (p100/p52), RelA (p65), RelB, and c-Rel^5^,^6^. Those NF- κB proteins dimerize with each other to form homo- and hetero-dimers and can modulate diverse biological responses, such as phagocytosis and apoptosis, by regulating target gene transcription^4^,^7^. However, NF-κB/Rel proteins are normally bound with inhibitor of κBs (IκBs) as an inactive form^8^, and can only be activated by phosphorylation of IκBs, which includes IκBa, IκBβ, IκBγ, IκBε, IκBζ, and BCL3 in the mammals^9^. Moreover, the pre-formed NF-κB-DNA complex during gene transcription can also be dissociated rapidly by free nuclear IκBs, attenuating the immune responses of the host^9^. The interaction between NF-κB proteins and IκBs is therefore decisive in maintaining homeostasis of host and are found rigorously modulated during challenge at multiple level, including transcriptional and post-transcriptional ones^4^,^5^,^6^,^9^.

miRNAs are an important class of short endogenous single-stranded non-coding RNAs (~22 nt in length) which could regulate gene expression at post-transcriptional level^10^. Since first found in *Caenorhabditis elegans*, more than 35,828 mature miRNAs have been identified so far in over 223 species^11^. Though structured similarly, miRNAs diversify greatly in their function. And almost all biological processes could be modulated by miRNAs, especially those immune-related processes^12^,^13^. For example, it was found that miRNAs induced after pathogen challenge could repress the synthesis and release of cytokines while other immune-related events such as phagocytosis, migration could also be modulated by those regulators^14^,^15^,^16^. Recently, miRNA-mediated modulation were likewise observed in multiple immune-related pathways, including NF-κB pathway^17^. For instance, miR-199a, a miRNA down-regulated in endometriosis, was proved to inhibit the IκB kinase β in embryonic stem cells and suppress the NF-κB pathway activation and interleukin-8 expression afterward^18^. Some other miRNAs such as miR-146a, miR-155, miR-181b and miR-21 were also annotated as regulators of NF-κB pathway^17^. Although mass of reports have revealed the interaction between miRNAs and NF-κB pathway in mammals, less is investigated in invertebrates^17^.

As an important intertidal bivalve, oyster *Crassostrea gigas* suffers continuously from hash environments and surrounding pathogens^19^. A robust immune response to fast eliminate invaded bacteria is therefore greatly needed^20^,^21^,^22^. With the release of genome information, oysters have been gradually regarded as a model in investigating invertebrate immune system with some components of NF-κB pathway characterized in the past decades, including one Rel and three IκBs^23^,^24^,^25^,^26^. Meanwhile, more than fifty immune-responsive miRNAs have been identified in *C. gigas*, among which cgi-miR-2d (Supplementary Fig. S1) was predicted as a modulator of CgIκB2^27^. The purposes of the present study were therefore to (1) survey the phagocytic changes of oyster haemocytes after *Vibrio splendidus* challenge, (2) revise the phylogeny of cgi-miR-2d, (3) investigate the interaction between of CgIκBs and cgi-miR-2d during challenge, and (4) reveal the modulation on haemocyte phagocytosis by cgi-miR-2d and hopefully provide new hints for the miRNA-mediated immunomodulation mechanism in oysters.

## Results

### Changes in haemocyte phagocytosis and CgIκBs expression during *V. splendidus* stimulation

**Figure 1:**
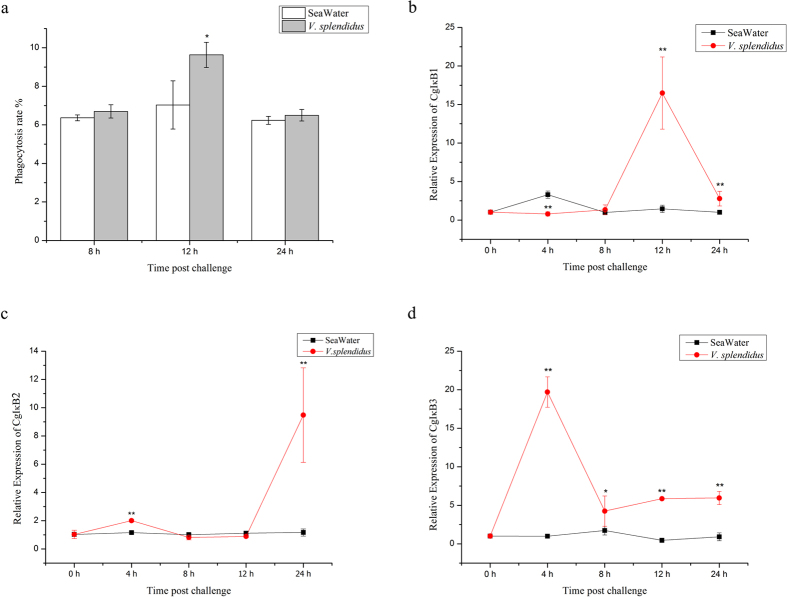
Alternations of haemocyte phagocytosis rate and CgIκBs expression levels during challenge. The phagocytosis rates of oyster haemocytes at 8 h, 12 h and 24 h after *V. splendidus* challenge were determined using flow cytometry (**a**). Expression levels of CgIκB1 (**b**), CgIκB2 (**b**) and CgIκB3 (**c**) during infection were also investigated by qRT-PCR. Significant differences were marked with asterisk “*” if *p* < 0.05 or “**” if *p* < 0.01.

The phagocytosis rate of oyster haemocytes was determined at 8 h, 12 h and 24 h post *V. splendidus* challenge. As a result, it remained unchanged at 8 h and 24 h post stimulation and increased significantly at 12 h (9.63% in challenge group *verse* 7.03% in seawater control group, *p* < 0.01) (Fig. 1a).

The expression level of CgIκB1 in oyster haemocytes decreased at 4 h post challenge yet recuperated afterward at 8 h. A transcriptional summit of CgIκB1 was then observed at 12 h post challenge (*p* < 0.01) (Fig. 1b). On the contrary, the CgIκB2 transcripts were found up-regulated robustly at 4 h post *V. splendidus* injection (2.00-fold of that in the control group, *p* < 0.01) and decreased at 8 h (Fig. 1c). No significant changes in CgIκB2 mRNA level were observed at 12 h until it ascended markedly at 24 h post injection, which reached 9.48-fold of that in control group at 0 h (*p* < 0.01) (Fig. 1c). The expression level of CgIκB3 also peaked at 4 h post challenge, and kept at a relatively higher level from 8 h to 24 h (*p* < 0.01) (Fig. 1d).

### Expression alternation of cgi-miR-2d during *V. splendidus* challenge

**Figure 2:**
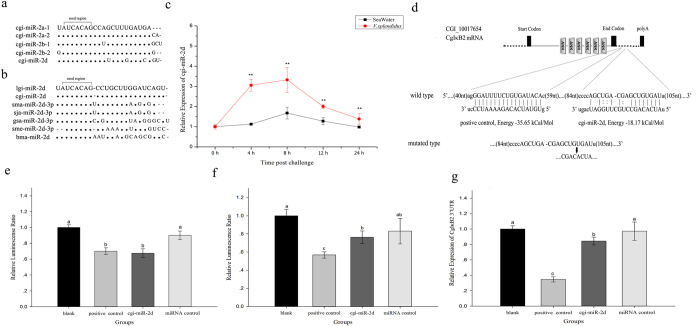
Interaction between cgi-miR-2d and CgIκB2 *in vitro*. The sequence similarity of members in oyster miR-2 family (including cgi-miR-2d) was illustrated by Cluster X (**a**). Nucleotide diversity could be found in miR-2d from *Lottia gigantean* (lgi), *Schistosoma mansoni* (sma), *Schistosoma japonicum* (sja), *Gyrodactylus salaris* (gsa), *Schmidtea mediterranea* (sme) and *Brugia malayi* (bma) while cgi-miR-2d was highly conserved with that in *L. gigantean* (**b**). The expression alternations of cgi-miR-2d were surveyed during *V. splendidus* challenge by qRT-PCR (**c**). Target genes of cgi-miR-2d were searched globally by miRanda and a binding site was found at CgIκB2 3′UTR (**d**). Luciferase reporter assay was subsequently conducted using wild type 3′UTR (**e**) or mutated type 3′UTR (**f**). The relative expression level of CgIκB2 3′UTR in cells transfected with wild type 3′UTR were also surveyed at 24 h post transfection (**g**). Significant differences were marked with letters (a, b, c *etc.*) if *p* < 0.05 or “**” if *p* < 0.01.

Five members of miR-2 family in oyster were first subjected to miRBase (http://www.mirbase.org) in search of homologues and were renamed subsequently according to their sequence similarity (Table 1). Consequently, a remarkable nucleotide similarity was observed among oyster miR-2 family members (Fig. 2a). Meanwhile, there was a great diversity within homologues of miR-2d from different organisms (Table 2) where cgi-miR-2d was highly conserved with that from *Lottia gigantean* (Fig. 2b). Moreover, all miR-2d were found derived from the 3′ arm of their precursor.

The expression changes of cgi-miR-2d during *V. splendidus* challenge were investigated subsequently. As a result, cgi-miR-2d transcripts were found increased rapidly after the challenge and peaked at 8 h post injection (3.32-fold of that in the control group, *p* < 0.01) (Fig. 2c). Though descended afterward, they still maintained at 1.41-fold of that in seawater control group (*p* < 0.01). No significant changes of the cgi-miR-2d transcripts were observed in seawater control group during the experiment (Fig. 2c).

### The interaction between cgi-miR-2d and CgIκB2 *in vitro*

CgIκB2 3′UTR (291 bp in length) containing putative binding site of cgi-miR-2d (from 84 to 105 nt, Fig. 2d) was first cloned using gene-specific primer and inserted subsequently into the psiCHECK-2 vector (designated as wild type vector) for luciferase reporter assays in HEK293T cells. At 24 h post transfection, the relative luminescence ratio in each group was detected and a significant decrease was observed in cgi-miR-2d group (32.40%) in comparison with that in blank or miRNA control group (*p* < 0.05) (Fig. 2e). Similarly, the decrease in relative luminescence ratio could also be observed in positive control group (binding site from 40 to 59 nt, *p* < 0.05) (Fig. 2d,e). To further verify the binding specificity of cgi-miR-2d, mutation was made on the CgIκB2 3′UTR (designated as mutated type) where was complementary with seed region of cgi-miR-2d (Fig. 2d). Consequently, no significant changes were observed in relative luminescence ratio of cgi-miR-2d group while it decreased significantly in positive control group (*p* < 0.05) (Fig. 2f).

The relative expression level of CgIκB2 3′UTR was subsequently measured in cells transfected with wild type vector by quantitative real-time PCR (qRT-PCR). Consistently, CgIκB2 3′UTR decreased significantly in both cgi-miR-2d group (0.84-fold of that in miRNA control group, *p* < 0.05) and positive control group (0.35-fold of that in miRNA control group, *p* < 0.05) when compared to that in blank or miRNA control group (Fig. 2g).

### The interaction between cgi-miR-2d and CgIκB2 *in vivo* and modulation on phagocytosis rate and apoptosis rate

**Figure 3:**
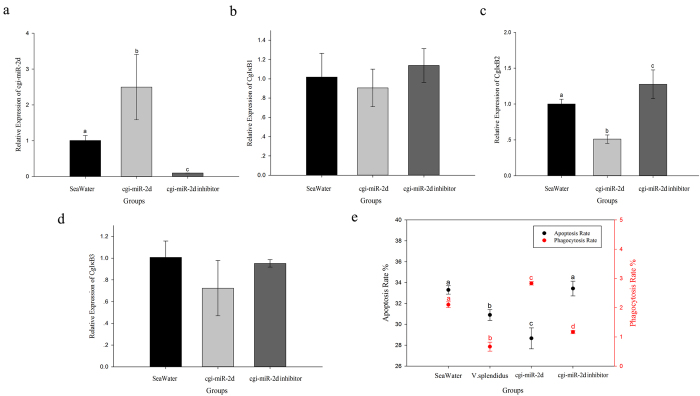
Alternations of CgIκBs mRNA and haemocyte phagocytosis and apoptosis rate in gain- and loss-of-function assay of cgi-miR-2d *in vivo*. Gain- and loss-of-function assay were conducted *in vivo* by injection of cgi-miR-2d mimics or inhibitors. cgi-miR-2d transcripts were firstly surveyed at 24 h post injection by qRT-PCR (**a**). Expression alternations of CgIκB1 (**b**), CgIκB2 (**c**) and CgIκB3 (**d**) were also investigated. Haemocyte phagocytosis and apoptosis rate were invesitigated using flow cytometry simultaneously (**e**). Significant differences were marked with different letters (a, b, c *etc.*) if *p* < 0.05.

Gain- and loss-of-function assay of cgi-miR-2d were subsequently conducted *in vivo* by injecting cgi-miR-2d mimics and inhibitors into oysters. The cgi-miR-2d transcripts were first investigated at 24 h post injection. Consequently, a significant increase of cgi-miR-2d transcripts was observed in cgi-miR-2d group (2.49-fold of that in seawater group, *p* < 0.05) (Fig. 3a) while they decreased robustly when cgi-miR-2d inhibitors were injected (0.10-fold of seawater control group, *p* < 0.05) (Fig. 3a). However, no significant changes of CgIκB1 or CgIκB3 transcripts were observed in either group at 24 h post injection (Fig. 3b,d). Comparatively, CgIκB2 transcripts decreased simultaneously in cgi-miR-2d group (0.51-fold of seawater group, *p* < 0.05) while increased significantly to 1.27-fold in cgi-miR-2d inhibitor group when compared with seawater group (*p* < 0.05) (Fig. 3c).

Alternations in phagocytosis and apoptosis rate of oyster haemocytes were also surveyed. It turned out that haemocyte phagocytosis rate increased after cgi-miR-2d overexpression (2.83%, *p* < 0.05) and decreased when cgi-miR-2d was repressed by its inhibitor (1.16%, *p* < 0.05), in comparison with that in seawater group (2.10%, Fig. 3e). In addition, a significant decrease of phagocytosis rate was observed at 24 h after *V. splendidus* challenge (Fig. 3e). The apoptosis rate of haemocytes was down-regulated significantly (28.57%, *p* < 0.05) after gain-of-function assay of cgi-miR-2d whereas it remained unchanged after cgi-miR-2d inhibition (Fig. 3e). The apoptosis rate of haemocytes in bacteria challenge group, however, decreased remarkably at 24 h (*p* < 0.05) (Fig. 3e).

### Haemocyte phagocytic and apoptotic changes after CgIκB2 knock-down assay *in vivo*

**Figure 4:**
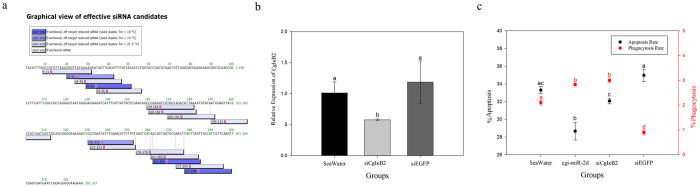
Alternations of CgIκB2 mRNA and haemocyte phagocytosis and apoptosis rate after knock-down of CgIκB2 *in vivo*. Konck-down of CgIκB2 was conducted *in vivo* by injection of dsRNA designed by http://sidirect2.rnai.jp/ with default settings (**a**). Expression changes of CgIκB2 mRNA were detected by qRT-PCR at 24 h post the injection (**b**). Phagocytosis and apoptosis rate of haemocytes were also surveyed (**c**). Significant differences were marked with different letters (a, b, c *etc.*) if *p* < 0.05.

DsRNA of CgIκB2 was synthesized *in vitro* using a fragment from CgIκB2 coding region which was unique in genome and injected into oysters for knock-down assay *in vivo* (Fig. 4a, Supplementary Fig. S2). The expression level of CgIκB2 in siCgIκB2 group was surveyed at 24 h post injection and was found declined remarkably (0.48-fold of that in siEGFP group, *p* < 0.05) (Fig. 4b). Correspondingly, the phagocytosis rate increased significantly in siCgIκB2 group when compared with that in siEGFP group (3.00% *verse* 0.90%, *p* < 0.05) (Fig. 4c). And the haemocyte apoptosis rate decreased after the interference of CgIκB2 in comparison with that in siEGFP control group (*p* < 0.05) (Fig. 4c).

## Discussion

Phagocytosis against bacteria has been regarded as a fundamental event in host immune process and contributes greatly to the homeostasis of organism during pathogen challenge^21^. It has been observed that phagocytosis of molluscan haemocytes could also be rapidly triggered after stimulation and dedicate to the fast elimination of invaded microbes^22^,^28^,^29^. In the present study, the phagocytosis rate of oyster haemocytes also increased significantly at 12 h post *V. splendidus* challenge (Fig. 1a) and declined afterward. The spontaneous alternation in haemocyte phagocytosis highlighted the intense immune response inside the oysters and indicated the rigorous modulation beneath.

Within mass of immune-related pathways, the NF-κB pathway has been well investigated as a global regulator of immune response including phagocytosis, where IκB genes are regarded as hallmarks^4^. Here, the expression levels of three CgIκBs in haemocytes were also surveyed during challenge in reflection of NF-κB activation. As a result, three CgIκBs were rigorously modulated during challenge (Fig. 1b–d) with different expression pattern, which was similar with previous findings^25^,^26^, demonstrating the dynamic involvement of NF-κB pathway in immune response of mollusk as well as the functional distinctions among CgIκBs^30^. Moreover, an opposite alternation pattern was observed between the CgIκB2 transcripts and haemocyte phagocytosis (Fig. 1a,c). Given the interaction between phagocytosis and NF-κB pathway in mammals, we deduced audaciously that oyster phagocytosis could also be modulated by CgIκB2. Correspondingly, the phagocytosis rate of oyster haemocytes increased significantly in CgIκB2 knock-down assay *in vivo* (Fig. 4a,b), confirming our speculations above. Paradoxically, CgIκB2 remained at basal level at 12 h post challenge, suggesting the existence of post-transcription regulation. Within the expectation, cgi-miR-2d, a putative regulator of CgIκB2, was found up-regulated markedly during bacteria challenge (Fig. 2c). The interaction between cgi-miR-2d and CgIκB2 was then verified both *in vitro* and *in vivo*.

To date, mass of miRNAs has been identified and proved crucial in diverse biological processes, especially in immune response^31^. It was also suggested that majority of miRNAs could regulate target genes post-transcriptionally by binding to their 3′UTR region^17^,^32^. The putative modulation by cgi-miR-2d was first confirmed *in vitro* by CgIκB2 3′UTR luciferase reporter assay in HEK293T cells (Fig. 2d). Consequently, an intense depression of relative luminescence ratio was observed when cgi-miR-2d was co-transfected with wild type CgIκB2 3′UTR (Fig. 2e) while it remained unchanged when the binding site of cgi-miR-2d at 3′UTR was mutated (Fig. 2f). Besides, some reports have also found that the transcripts of target genes could be degraded partly by miRNA in imprecise complementation with their 3′UTR^33^, or completely when miRNAs were in complete complementation^34^,^35^. Herein, similar results could also be observed where CgIκB2 3′UTR transcripts decreased in both positive control and cgi-miR-2d group (Fig. 2g), reconfirming the interaction between CgIκB2 3′UTR and cgi-miR-2d *in vitro*. The interaction between cgi-miR-2d and CgIκB2 was then verified *in vivo* by gain- and loss-of- function assay of cgi-miR-2d. Consistently, the expression level of CgIκB2 decreased significantly during gain-of-function assay of cgi-miR-2d *in vivo*, and increased when endogenous cgi-miR-2d was repressed by its inhibitor (Fig. 3a,c). Meanwhile, no significant changes of CgIκB1 or CgIκB3 transcripts were observed in gain- and loss-of-function assay of cgi-miR-2d (Fig. 3b,d). Collectively, those results confirmed the interaction between cgi-miR-2d and CgIκB2, which might dedicate crucially in modulating host immune response.

**Figure 5:**
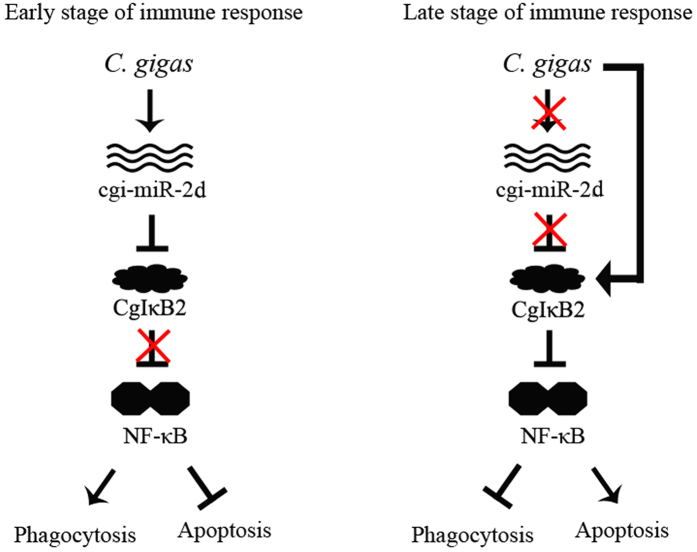
Proposed role of cgi-miR-2d in oyster immune response by targeting CgIκB2. Given the expression changes of cgi-miR-2d and CgIκB2, it was suggested that CgIκB2 could be modulated post-transcriptionally by up-regulated cgi-miR-2d at the early stage of *V. splendidus* infection and led to the inhibition of NF-κB pathway activation as well as the promotion of haemocyte phagocytosis. At the late stage of *V. splendidus* challenge, expression of cgi-miR-2d was repressed while that of CgIκB2 was up-regulated to disadvantage both NF-κB pathway and haemocyte phagocytosis. Moreover, apoptosis could also be regulated by cgi-miR-2d, which was conserved among miR-2 family yet not dominant during *V. splendidus* challenge, indicating the existence of other unknown modulatory pathways.

As mentioned, massive reports in mammals have revealed the interaction between NF-κB pathway and phagocytosis^36^,^37^,^38^. Recently, report in *Apostichopus japonicus* has also depicted the participation of miRNAs in haemocytes phagocytosis^12^. Given that CgIκBs could repress NF-κB activation *in vitro*^25^,^26^, the interaction between cgi-miR-2d and CgIκB2 was therefore supposed to augment haemocyte phagocytosis by modulating NF-κB pathway. Unexpectedly, a significant increase of phagocytic rate was observed in cgi-miR-2d overexpression group (Fig. 3c), which was similar with that in CgIκB2 knock-down group (Fig. 4c). And the increase caused by cgi-miR-2d could also be reversed by cgi-miR-2d inhibitors (Fig. 3c), accompanying with increase of CgIκB2 transcripts. Among the numerous miRNAs identified from diverse species^39^, miR-2 family was found expressed exclusively in invertebrates and could promote cell survival^40^. In the meantime NF-κB pathway has also been well-known in anti-apoptosis in either oysters (Fig. 4c) or mammals^7^,^37^,^38^. Thence, alternations on haemocyte apoptosis rate were surveyed simultaneously. Accordingly, it decreased significantly after gain-of-function assay of cgi-miR-2d *in vivo* and increased when cgi-miR-2d was inhibited (Fig. 4c). However, the apoptosis rate decreased remarkably after *V. splendidus* challenge, suggesting a more complicated modulation network during stimulation. Nevertheless, these results verified the interaction between cgi-miR-2d and CgIκB2 and depicted their contribution on the phagocytosis rate of haemocytes. Given to the expression changes of cgi-miR-2ad and CgIκB2, their interaction might also dedicate importantly to oysters immune response during bacteria challenge (Fig. 5)^27^ as well as the oysters’ thriving in intertidal regions.

## Materials and Methods

### Oyster culture, bacteria challenge and sample collection

Oysters *C. gigas* (averaging 150 mm in shell length, 70 mm in width) engaged in this experiment were collected from a local farm in Qingdao, China. A narrow notch was sawed in the oyster shell where was close to the adductor muscle for subsequent injection^20^. All oysters were then acclimatized in aerated sea water (about 20 °C) for two weeks before use.

A total of 30 oysters were employed for bacteria stimulation to investigate expression changes of cgi-miR-2d identified previously^27^. Briefly, oysters in PBS control group and *V. splendidus* challenge group were injected with 100 μL of phosphate buffered saline (PBS, 0.14 mol L^−1^ sodium chloride, 3 mmol L^−1^ potassium chloride, 8 mmol L^−1^ disodium hydrogen phosphate dodecahydrate, 1.5 mmol L^−1^ potassium phosphate monobasic, pH 7.4) and 100 μL suspension of alive *V. splendidus* strain^41^ (1 × 10^7^ CFU mL^−1^ in PBS), respectively. At 12 h later, haemocytes from five oysters in each group were collected from cardiocoelom by centrifugation at 800 *g*, 4 °C for 10 minutes and pooled together for subsequent miRNA extraction and qRT-PCR analysis of cgi-miR-2d. Another 360 oysters were also employed for bacteria challenge. Similarly, oysters in seawater control group and *V. splendidus* challenge group were stimulated with 100 μL sterile seawater and 100 μL suspension of live *V. splendidus* strain (1 × 10^7^ CFU mL^−1^ in sterile seawater), respectively. Haemocytes from five oysters in each group were collected at 0, 4, 8, 12 and 24 h post injection, and pooled together for subsequent RNA extraction and qRT-PCR analysis of CgIκBs. Haemocytes from another five individuals were also sampled for quantitative analysis of cgi-miR-2d. Additional five oysters in each group were sampled likewise at 8, 12 and 24 h post injection for the analysis of haemocyte phagocytic rate.

All trials were conducted with three biological replicates.

### RNA isolation, cDNA synthesis and SYBR Green fluorescent qRT-PCR

RNA isolation and cDNA synthesis were conducted using methods in previous reports^42^. The SYBR Green fluorescent qRT-PCR was carried out in an ABI 7500 Real-time Thermal Cycler according to the manual (Applied Biosystems). The gene-specific primers were designed according to its cDNA sequences and listed on Table 3. Briefly, a reaction mix with 5 μL of 2× SYBR Green Master Mix (Takara), 2 μL of the diluted cDNA templates, 0.2 μL of each primers (10 mmol L^−1^), 0.2 μL ROX Reference Dye II and 2.4 μL of DEPC water was used to amplify corresponding genes. The elongation factor (EF) gene^43^ was used as an internal control for the expression analysis of oyster CgIκBs.

Total miRNAs were extracted using PureLink miRNA Isolation Kit (Invitrogen) according to the manufacture’s protocol. The synthesis of cDNA was conducted using miScript II RT (Qiagen) with miRNA extracted above at 37 °C for 1 h and terminated by heating at 95 °C for 5 min. The cDNA mix obtained was diluted with the addition of 200 μL RNase-free water before use. The SYBR green fluorescent qRT-PCR was carried out in a total volume of 25.0 μL, containing 12.5 μL of 2× miScript SYBR Green PCR Master Mix (Qiagen), 2.5 μL of diluted cDNA, 2.5 μL of each primers (10 mmol L^−1^), and 5.0 μL of RNase-free water. All primers were listed in Table 3, and the 5S rRNA was used as internal control.

All data were given in terms of relative mRNA or miRNA expression using the 2^−ΔΔCt^ method^44^.

### Target prediction of cgi-miR-2d and 3′UTR luciferase reporter assay

The cgi-miR-2d mimics which would alter into single strand *in vivo* and be identical with endogenous cgi-miR-2d were synthesized by GenePharma. A positive miRNA mimics with binding capability to CgIκB2 3′UTR (from 40 to 59 nt) was also synthesized. miRNA control originated from *C. elegans* and could not target any oyster genes or mimic any oyster miRNAs was as well employed. Cgi-miR-2d inhibitors which were in complete complementation with cgi-miR-2d and could sequester it by binding were synthesized for loss-of-function assay. All RNA was diluted at a final concentration of 20 μmol L^−1^ using DEPC water before use. These RNA sequences were listed on Table 3.

Target prediction of cgi-miR-2d was conducted by miRanda using 3′UTR sequences deduced from oyster genome information^19^. The wild type or mutated type of CgIκB2 3′UTR was cloned with gene-specific primers (Table 3) and inserted into psiCHECK-2 vector (Promega) for subsequent luciferase reporter assay with methods described previously^15^. Briefly, a total of 1 × 10^5^ HEK293T cells were seeded into each well of 48-well plates and cultured overnight before transfection. Cells in positive control, cgi-miR-2d and miRNA control were then transfected with a mixture of 100 ng luciferase reporter plasmid (extracted by Tiangen EndoFree Maxi Plasmid Kit) and 5 pmol positive control or cgi-miR-2d mimics or miRNA control using Lipofectamine 2000 reagent (Invitrogen) according to the protocol. Cells transfected merely with recombined vector were employed as blank group. The detailed information was listed on Supplementary Table S1.

The luciferase activities in those groups were measured at 24 h post transfection according to the manufacturer’s instruction using Dual-Luciferase Reporter Assay System kit (Promega). Briefly, cells in each well were firstly lysed using Passive Lysis Buffer provided by the kit. A total of 100 μL LARII was then added into 20 μL cell lysates to detect the firefly luciferase activity using luminometer. And a volume of 100 μL Stop & Glo Reagent was added into the mixture to measure renilla luciferase activity. Cells transfected with wild type 3′UTR were also harvested for quantitative real-time PCR of CgIκB2 3′UTR with GAPDH as the internal control. Each trial was conducted with three replicates.

### Gain- and los-of-function assay of cgi-miR-2d *in vivo*

A number of 90 oysters were employed for gain- and loss-of-function assay and randomly divided into three groups (designated as seawater, cgi-miR-2d and cg-miR-2a inhibitor group), receiving an injection of 100 μL sterile seawater, 2.5 nmol cgi-miR-2d mimics (in 100 μL sterile seawater) and 2.5 nmol cgi-miR-2d inhibitors (in 100 μL sterile seawater), respectively. Haemocytes from five oysters in each group were collected afterward at 24 h post injection to survey expression changes of CgIκBs. The phagocytosis and apoptosis rate of haemocytes were also surveyed at the same time using another five oysters. Oysters challenged with *V. splendidus* for 24 h (designated as *V. splendidus* group) were also employed and subjected for phagocytosis and apoptosis assay. Each trial was conducted with three replicates.

### CgIκB2 knock-down assay *in vivo*

DsRNA synthesis was conducted using method described in previous reports^45^. Briefly, a fragment from CgIκB2 coding region (85 nt to 474 nt) which was unique among oyster coding genes was firstly cloned and subjected to siDirect2 (http://sidirect2.rnai.jp/) for siRNA prediction. A pair of T7 promoter linked primer was then employed for *in vitro* transcription of CgIκB2 dsRNA. A DNA fragment (657 bp) from pEGFP-N1 vector (Clontech) was also cloned to synthesize control dsRNA^45^.

A total of 90 oysters were employed and randomly divided into three groups for subsequent knock-down experiment. Oysters in seawater group, siCgIκB2 group and siEGFP group were injected with 100 μL sterile seawater, 100 μg dsRNA of IκB (in 100 μL sterile seawater) and 100 μg dsRNA of EGFP (in 100 μL sterile seawater), respectively, and the haemocytes were sampled from cardiocoelom at 24 h later to detect expression changes of CgIκB2. The haemocytes from another five oysters in each group were also collected for phagocytic and apoptosis detection. All the trials were conducted with three parallel replicates.

### Determination of haemocyte phagocytosis and apoptosis rate

Phagocytosis rate was determined using the method modified from previous report^20^. In brief, *V. splendidus* cultured at 16 °C overnight was labeled by FITC (Sigma) and diluted to 10^8^ cells mL^−1^ for later use. Oyster haemocytes collected freshly with acid citrate-dextrose anticoagulant agent (22 g L^−1^ Sodium Citrate, 8 g L^−1^ citric acid, 24.5 g L^−1^ glucose, pH 7.4) were resuspended in L15 medium (Gibco) to a final concentration of 2 × 10^6^ cells mL^−1^ before the incubation with the same volume of FITC-labeled *V. splendidus* for 60 min. The incubated haemocytes were then washed for three times with L15 medium to remove extracellular bacteria. After a recollection by centrifugation at 800 *g*, 4 °C for 5 min, haemocytes were subjected to flow cytometry (BD Biosciences) to investigate phagocytosis rate.

Haemocyte apoptosis rate was measured using the Annexin V-FITC Detection Kit (Beyotime). In brief, 200 μL of diluted haemocytes were incubated firstly with 5 μL of Annexin V-FITC in the dark at room temperature for 10 min and then with 10 μL propidium iodide for 5 min. Haemocytes were also subjected to flow cytometry for apoptosis rate detection after the wash and recollection.

### Statistical analysis

All data were given as means ± S.D. One-way analysis of variance (one-way ANOVA) followed by a multiple comparison (LSD method) was used subsequently to determine difference. Asterisks (^*^ if *p* < 0.05, ^**^ if *p* < 0.01) or different letters (a, b, c *etc*. if *p* < 0.05) were marked on the top of bar to indicate significant difference.

## Additional Information

**How to cite this article**: Chen, H. *et al*. An invertebrate-specific and immune-responsive microRNA augments oyster haemocyte phagocytosis by targeting CgIκB2. *Sci. Rep.* **6**, 29591; doi: 10.1038/srep29591 (2016).

## Supplementary Material

Supplementary Information

## Figures and Tables

**Table 1:** Homologues of miR-2 family in oyster.

Previous ID	Present ID	Sequence (5′-3′)
cgi-miR-2e	cgi-miR-2a-1	UAUCACAGCCAGCUUUGAUGA
cgi-miR-2d	cgi-miR-2a-2	UAUCACAGCCAGCUUUGAUGACA
cgi-miR-2b	cgi-miR-2b-1	UAUCACAGCUAGCUUUGAUGAGCU
cgi-miR-2c	cgi-miR-2b-2	GAUCACAGCCAGCUUUGAUGAG
cgi-miR-2a	cgi-miR-2d	UAUCACAGCCUGCUUGGAUCAGU

**Table 2:** Sequences of miR-2d from different species.

Specie	ID	Sequence (5′-3′)
*Gyrodactylus salaris*	gsa-miR-2d-3p	UAUCACAGCCGUGCUUUAAGGGCUU
*Brugia malayi*	bma-miR-2d	UAUCACAGAAUUGAUGCAGCGAGC
*Schistosoma mansoni*	sma-miR-2d-3p	UAUCACAGUCCUGCUUAGGUGA
*Schistosoma japonicum*	sja-miR-2d-3p	UAUCACAGUCCUGCUUAGGUGA
*Lottia gigantean*	lgi-miR-2d	UAUCACAGCCUGCUUGGAUCAGU
*Schmidtea mediterranea*	sme-miR-2d-3p	UCACAGCCAAAUUUGAUGUCC
*Crassostrea gigas*	cgi-miR-2d	UAUCACAGCCUGCUUGGAUCAGU

**Table 3:** Primers and RNAs used in this study.

Category	Primer and RNA name	Sequence (5′-3′)
RNAs	cgi-miR-2d mimics	UAUCACAGCCUGCUUGGAUCAGU UGAUCCAAGCAGGCUGUGAUAUU
	Positive control	GUGUAUCACAGAAAAUCCCU GGAUUUUCUGUGAUACACUU
	miRNA control	UUCUCCGAACGUGUCACGUTT ACGUGACACGUUCGGAGAATT
	cgi-miR-2d inhibitor	ACUGAUCCAAGCAGGCUGUGAUA
UTR clone primers	CgIκB2_UTRclone_F	TATTACAATCTGGTGCCGATGTGA
	AP-dT	GGCCACGCGTCGACTAGTACT_17_
	CgIκB2_mutate_F	AGCTGACGACGACACTATGGCTGATTG
	CgIκB2_mutate_R	ACAATCAGCCATAGTGTCGTCGTCAGC
Recombination primers	CgIκB2 _ XhoI	CTCGAGAGAAAGGACTGCCGAA
	CgIκB2 _ NotI	GCGGCCGCTTTAGACTTGTC
RNAi clone primers	siCgIκB2 _basic_F	AGACCCATGCAAAATCTGGAC
	siCgIκB2 _basic_R	CTTCTTCCTCCTCCGATTCATC
	siCgIκB2_T7_F	TAATACGACTCACTATAGGGATC AGACCCATGCAAAATCTGGAC
	siCgIκB2_T7_R	TAATACGACTCACTATAGGGATC CTTCTTCCTCCTCCGATTCATC
	siEGFP_basic_F	CGACGTAAACGGCCACAAGT
	siEGFP_basic_R	CTTGTACAGCTCGTCCATGC
	siEGFP_T7_F	TAATACGACTCACTATAGGGA TCCGACGTAAACGGCCACAAGT
	siEGFP_T7_R	TAATACGACTCACTATAGGGATC CTTGTACAGCTCGTCCATGC
Real-Time primers	GAPDH_F	AGGTCGGTGTGAACGGATTTG
	GAPDH_R	TGTAGACCATGTAGTTGAGGTCA
	EF-F	AGTCACCAAGGCTGCACAGAAAG
	EF_R	TCCGACGTATTTCTTTGCGATGT
	CgIκB1 _RT_F	CCCTTCACATTGCCAGTAG
	CgIκB1 _RT_R	ATTGGGAGATGGGTGTTCT
	CgIκB2 _RT_F	CGAGTGATGAATCGGAGGAGG
	CgIκB2 _RT_R	CACACAATCAGCCAATCACAGC
	CgIκB3 _RT_F	ACCTCCCCTCCCTACAACCTCAGACT
	CgIκB3 _RT_R	CCTGGTGACATGGAATGGGCAACT
	cgi-miR-2d	TATCACAGCCTGCTTGGATCAGT
	rRNA_5s	CAAGGATGACACGCAAAT
