# Supplementary material for: An invertebrate-specific and immune-responsive microRNA augments oyster haemocyte phagocytosis by targeting CgIκB2
Source: Sci Rep. 2016 Jul 12;6:29591. doi: 10.1038/srep29591 (PMC4941716; doi:10.1038/srep29591)
Supplement: Supplementary Information [file srep29591-s1.pdf]

An invertebrate-specific and immune-responsive microRNA augments oyster haemocyte phagocytosis by targeting CgIκB2

Hao Chen[1,3], Zhi Zhou[1], Hao Wang[1], Lingling Wang[1*], Weilin Wang[1,3], Rui Liu[1], Limei Qiu[1], Linsheng Song[1, 2*]

[1] Key Laboratory of Experimental Marine Biology, Institute of Oceanology, Chinese Academy of Sciences, Qingdao 266071, China

[2] Key Laboratory of Mariculture & Stock enhancement in North China's Sea, Ministry of Agriculture, Dalian Ocean University, Dalian 116023, China

[3] University of Chinese Academy of Sciences, Beijing 100049, China

*** Corresponding author:**

Prof. Linsheng Song (lshsong@dlou.edu.cn)

Dalian Ocean University, 52 Heishijiao Street, Dalian 116023, China.

Tel: +86 411 84762652; Email address: lshsong@dlou.edu.cn

Prof. Lingling Wang (wanglingling@qdio.ac.cn)

Institute of Oceanology, Chinese Academy of Sciences, 7 Nanhai Street, Qingdao 266071, China.

Tel: +86 532 82898843; Email address: wanglingling@qdio.ac.cn

Table S1. Group and transfection information of the 3'UTR luciferase reporter assay

Group	Blank	Positive control	cgi-miR-2d	miRNA control
Recombined vector	100ng	100ng	100ng	100ng
Positive control		5 pmol		
Cgi-miR-2d mimics			5 pmol	
miRNA control				5 pmol

Table S1. Group and transfection information of the 3'UTR luciferase reporter assay conducted in the present study. A total of 100 ng recombined plasmid containing either wild type or mutated type CgIκB2 3'-UTR was transfected into cells of blank group. An additional of 5 pmol positive control mimics or cgi-miR-2d mimics or miRNA control were co-transfected with the plasmid into cells of positive control, cgi-miR-2d and miRNA control group, respectively.

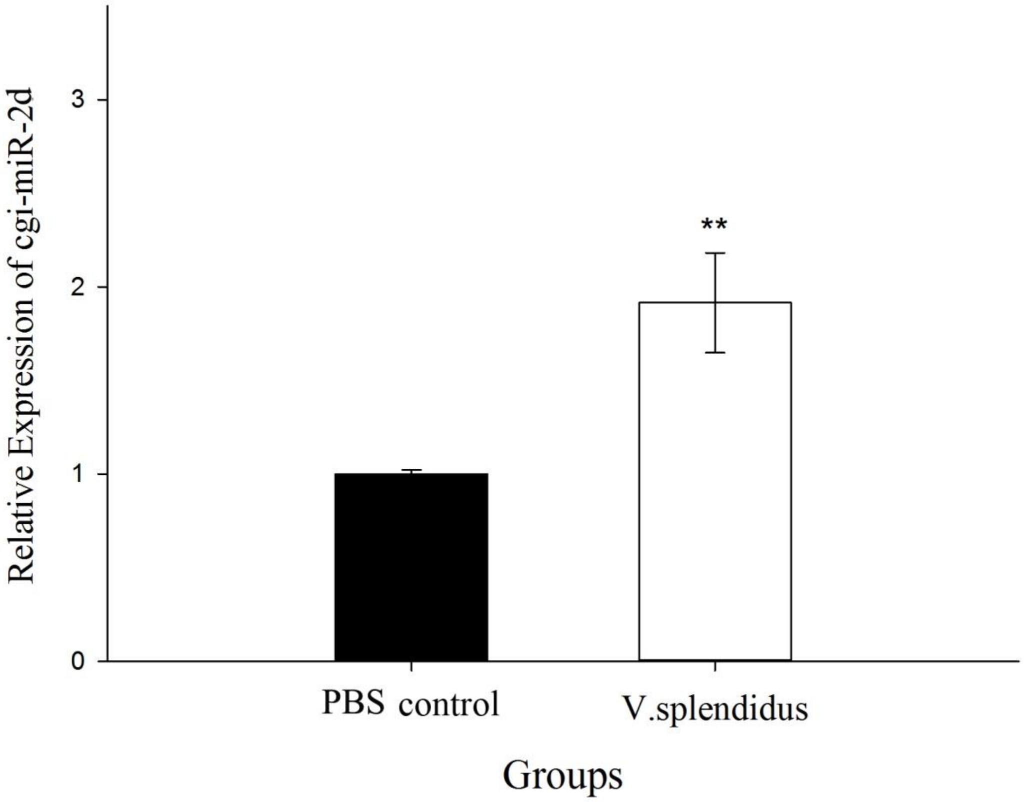

Figure S1. Alternations of cgi-miR-2d transcripts in challenge group at 12h compared to that in PBS control group.

A significant increase of cgi-miR-2d was observed in *V. splendidus* challenge group at 12 h post injection in comparison with that in PBS control group which was consistent with results reported by Zhou Zhi *et.al.*.

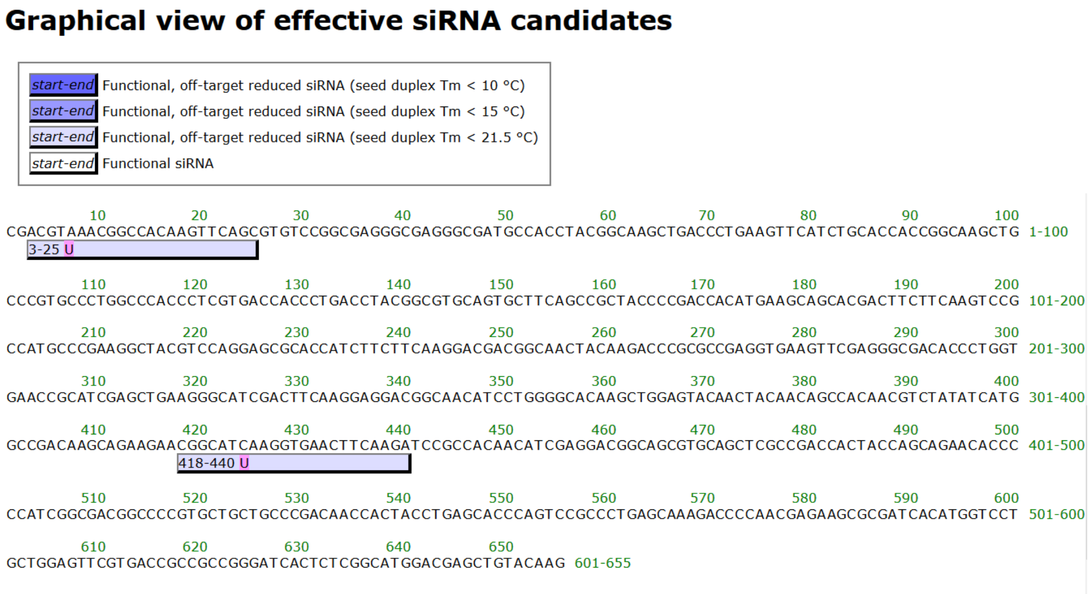

Figure S2. siRNA candidates of dsRNA of sequence from pEGFP-N1.

A DNA fragement of pEGFP-N1 was cloned to synthesize dsRNA control. The putative siRNA candidates of pEGFP-N1 dsRNA were predicted by http://sidirect2.rnai.jp/ with default settings and illustrated in color.
